# Cognitively unimpaired individuals with a low burden of Aβ pathology have a distinct CSF biomarker profile

**DOI:** 10.1186/s13195-021-00863-y

**Published:** 2021-07-27

**Authors:** Marta Milà-Alomà, Mahnaz Shekari, Gemma Salvadó, Juan Domingo Gispert, Eider M. Arenaza-Urquijo, Grégory Operto, Carles Falcon, Natalia Vilor-Tejedor, Oriol Grau-Rivera, Aleix Sala-Vila, Gonzalo Sánchez-Benavides, José Maria González-de-Echávarri, Carolina Minguillon, Karine Fauria, Aida Niñerola-Baizán, Andrés Perissinotti, Maryline Simon, Gwendlyn Kollmorgen, Henrik Zetterberg, Kaj Blennow, Marc Suárez-Calvet, José Luis Molinuevo, for the ALFA study

**Affiliations:** 1 Barcelonaβeta Brain Research Center (BBRC), Pasqual Maragall Foundation, Wellington 30, 08005 Barcelona, Spain; 2 IMIM (Hospital del Mar Medical Research Institute), Barcelona, Spain; 3 Universitat Pompeu Fabra, Barcelona, Spain; 4 Centro de Investigación Biomédica en Red de Fragilidad y Envejecimiento Saludable (CIBERFES), Madrid, Spain; 5 Centro de Investigación Biomédica en Red Bioingeniería, Biomateriales y Nanomedicina, Madrid, Spain; 6 Centre for Genomic Regulation (CRG), The Barcelona Institute for Science and Technology, Barcelona, Spain; 7 Department of Clinical Genetics, ERASMUS MC, Rotterdam, the Netherlands; 8 Servei de Neurologia, Hospital del Mar, Barcelona, Spain; 9 Servei de Medicina Nuclear, Hospital Clínic, Barcelona, Spain; 10 Roche Diagnostics International Ltd., Rotkreuz, Switzerland; 11 Roche Diagnostics GmbH, Penzberg, Germany; 12 Department of Psychiatry and Neurochemistry, Institute of Neuroscience and Physiology, University of Gothenburg, Mölndal, Sweden; 13 Clinical Neurochemistry Laboratory, Sahlgrenska University Hospital, Mölndal, Sweden; 14 Department of Neurodegenerative Disease, UCL Institute of Neurology, Queen Square, London, UK; 15 UK Dementia Research Institute at UCL, London, UK; 16 Present address: H. Lundbeck A/S, Copenhagen, Denmark

**Keywords:** Preclinical, Alzheimer’s disease, CSF, Biomarkers, Subthreshold, Cognitively unimpaired

## Abstract

**Background:**

Understanding the changes that occur in the transitional stage between absent and overt amyloid-β (Aβ) pathology within the Alzheimer’s *continuum* is crucial to develop therapeutic and preventive strategies. The objective of this study is to test whether cognitively unimpaired individuals with a low burden of Aβ pathology have a distinct CSF, structural, and functional neuroimaging biomarker profile.

**Methods:**

Cross-sectional study of 318 middle-aged, cognitively unimpaired individuals from the ALFA+ cohort*.* We measured CSF Aβ42/40, phosphorylated tau (p-tau), total tau (t-tau), neurofilament light (NfL), neurogranin, sTREM2, YKL40, GFAP, IL6, S100B, and α-synuclein. Participants also underwent cognitive assessments, *APOE* genotyping, structural MRI, [^18^F]-FDG, and [^18^F]-flutemetamol PET.

To ensure the robustness of our results, we used three definitions of low burden of Aβ pathology: (1) positive CSF Aβ42/40 and < 30 Centiloids in Aβ PET, (2) positive CSF Aβ42/40 and negative Aβ PET visual read, and (3) 20–40 Centiloid range in Aβ PET. We tested CSF and neuroimaging biomarker differences between the low burden group and the corresponding Aβ-negative group, adjusted by age and sex.

**Results:**

The prevalence and demographic characteristics of the low burden group differed between the three definitions. CSF p-tau and t-tau were increased in the low burden group compared to the Aβ-negative in all definitions. CSF neurogranin was increased in the low burden group definitions 1 and 3, while CSF NfL was only increased in the low burden group definition 1. None of the defined low burden groups showed signs of atrophy or glucose hypometabolism. Instead, we found slight increases in cortical thickness and metabolism in definition 2.

**Conclusions:**

There are biologically meaningful Aβ-downstream effects in individuals with a low burden of Aβ pathology, while structural and functional changes are still subtle or absent. These findings support considering individuals with a low burden of Aβ pathology for clinical trials.

**Trial registration:**

NCT02485730

**Supplementary Information:**

The online version contains supplementary material available at 10.1186/s13195-021-00863-y.

## Background

Alzheimer’s disease (AD) has an initial preclinical stage characterized by brain deposits of amyloid-β (Aβ) and tau, without clinical manifestations [1–5]. Moreover, other pathophysiological processes occur in this preclinical stage, including microglial activation, synaptic dysfunction, neuronal injury, and vascular dysfunction [6–8]. All these processes can occur years or even decades before the onset of the first symptoms [5, 9, 10]. Researchers, clinicians, and stakeholders in the AD field have learnt that the chances to succeed are higher if prevention and treatment interventions targeting these processes are applied as early as possible.

Aβ pathology can be detected with cerebrospinal fluid (CSF) biomarkers and Aβ positron emission tomography (PET). The Aβ cut-offs typically used to define Aβ-positivity (Aβ+) or Aβ-negativity (Aβ-) are usually derived from symptomatic AD patients and are extremely useful for clinical diagnosis at these stages. However, whilst the Aβ biomarker values in symptomatic AD show a bimodal distribution, in the preclinical stage, there is a progressive accumulation of Aβ. Therefore, the thresholds for diagnostic classification may not be optimal to detect subtle Aβ pathology in Aβ- individuals [11].

Growing evidence indicates that these Aβ subtle changes are meaningful [12]: Longitudinal analyses showed that subthreshold Aβ PET levels predict cognitive decline and tau deposition [13, 14]. Interestingly, the strongest association between subthreshold Aβ and cognition is found to occur in the middle age group [15]. Moreover, the rate of change of Aβ deposition peaks with an intermediate Aβ load, suggesting the existence of a critical time window for Aβ-lowering interventions [16–19].

Understanding this transitional stage from completely absent to overt amyloid-β (Aβ) pathology is key for clinical trials focused at this early stage. Our aim was to determine whether individuals with a low burden of Aβ pathology have a distinct CSF and neuroimaging biomarker profile that distinguish them from those who are clearly Aβ-. We hypothesized that subtle Aβ pathology (even below the usual thresholds for PET Aβ positivity) is already associated with Aβ-downstream pathophysiological changes, and/or cerebral structural and functional changes. In a cross-sectional design, we studied cognitively unimpaired individuals of the ALFA+ cohort. In order to ensure robustness, we applied three different definitions of low burden of Aβ pathology, in two of them combining both CSF and PET Aβ.

## Methods

### Participants

This is a cross-sectional study conducted in the ALFA+ cohort, a nested longitudinal study of the ALFA (for ALzheimer’s and FAmilies) study [20]. The ALFA study aims at characterizing preclinical Alzheimer’s and comprises 2743 cognitively unimpaired individuals, aged between 45 and 75 years old and enriched for family history of AD and *APOE*-ε4 status.

Among the 381 ALFA+ participants with CSF biomarkers available, we initially excluded those categorized as Aβ-negative (CSF Aβ42/40 ratio > 0.071) but tau-positive (CSF p-tau > 24 pg/ml) (n =12) to focus our study on the Alzheimer’s *continuum*. From those, we included participants that also had Aβ PET and FDG PET available (n = 318). Structural MRI with automatic segmentation was available in a total of 303 participants.

### CSF collection, processing, and storage: CSF biomarker measurements

CSF samples were obtained by lumbar puncture following standard procedures [21, 22]. Total tau and phosphorylated tau measurements were performed using the electrochemiluminescence immunoassays Elecsys® Total-tau CSF and Phospho-Tau(181P) CSF on a fully automated cobas e 601 instrument (Roche Diagnostics International Ltd.) [23]. Amyloid-β 42 (Aβ42) [24], amyloid-β 40 (Aβ40), neurofilament light (NfL), neurogranin, soluble triggering receptor expressed on myeloid cells 2 (sTREM2), chitinase-3-like protein 1 (YKL40), glial fibrillary acidic protein (GFAP), and α-synuclein were measured with the prototype NeuroToolKit (Roche Diagnostics International Ltd.) on a cobas e 411 instrument. Interleukin 6 (IL6) and calcium-binding protein B (S100B) were measured with the prototype NeuroToolKit (Roche Diagnostics International Ltd.) on a cobas e 601 instrument. All the measurements were conducted at the Clinical Neurochemistry Laboratory, Sahlgrenska University Hospital, Mölndal, Sweden.

### Neuroimaging data acquisition

Participants underwent [^18^F]-FDG and [^18^F]-flutemetamol PET scans following a cranial computed tomography (CT) scan for attenuation correction on a Siemens Biograph64 mCT camera. For acquiring [^18^F]-flutemetamol PET scans, participants received an intravenous bolus dose of 185 MBq (range 104.25–218.3 MBq, mean ± SD: 191.75 ± 14.04) and PET data was acquired after a 90-min post-injection (mean ± SD: 90.15 ± 7.36 min). [^18^F]-FDG PET scans were acquired 45 min (mean ± SD: 45.69±4.67) post-injection of 185 MBq (range 181.3–222 MBq, mean ± SD: 200.83 ± 12.83 MBq). All PET data were acquired for 20 min, using 4 frames of 5 min. PET images were reconstructed in 4 frames × 5 min using 3D ordered subset expectation maximization (OSEM) algorithm by incorporating time of flight (TOF) and point spread function (PSF) modeling.

MRI scans were obtained with a 3T scanner (Ingenia CX, Philips, Amsterdam, Netherlands). The MRI protocol was identical for all participants and included a high-resolution 3D T1-weighted Turbo Field Echo (TFE) sequence (voxel size 0.75 × 0.75 × 0.75 mm, TR/TE: 9.90/4.6 ms, flip angle = 8°).

### Visual assessment

Qualitative assessments were done for T1-weighted MRI and [^18^F]-flutemetamol PET images. A trained radiologist validated the image quality of MRI scans as well as incidental findings. Aβ PET images were visually rated by a nuclear medicine physician as Aβ+ or Aβ- using standard clinical criteria as specified in the Summary of Product Characteristics of the tracer [25].

### [^18^F]-flutemetamol PET quantification

[^18^F]-flutemetamol PET processing was performed following a validated Centiloid pipeline [26] using SPM12 [27]. Centiloid values (CL) were calculated from the mean values of the standard CL target region (http://www.gaain.org/centiloid-project) using the transformation previously calibrated [27].

### [^18^F]-FDG PET quantification

Quantification of [^18^F]-FDG PET was done by calculating the standard uptake value ratio (SUV_r_) within the region of interest (ROI). All preprocessing steps were performed using SPM12.

SUV_r_ values were calculated within a ROI composite, referred to as Meta-ROI. This composite was created by identifying regions cited frequently in [^18^F]-FDG PET studies of AD and MCI patients by Landau et al. [28]. ROI composite consists of five sub-regions including right and left angular gyri, middle/inferior temporal gyrus, and bilateral posterior cingulate gyrus. Meta-ROI SUVr of each [^18^F]-FDG PET image was calculated by averaging the SUVr over all the voxels involved in the Meta-ROI composite.

### Structural MRI quantification

T1-weighted images were automatically segmented and cortical thickness was measured in the regions from the Desikan-Killiany cortical atlas using Freesurfer version 6.0 [29]. Segmentation results were visually quality controlled by an expert. The cortical AD signature was then estimated for each subject based on the thickness of the following areas: entorhinal, inferior temporal, middle temporal, and fusiform. The signature was calculated as the mean thickness across these regions weighted by their surface area, as previously proposed [18, 30]. Additionally, we selected a set of individual regions to be included in the analysis, namely the banks of the superior temporal sulcus (bankssts), precuneus, and hippocampus, all sensitive to AD pathology [18, 31, 32].

### Low burden of Aβ pathology definitions

Aβ pathology was assessed using both CSF Aβ and Aβ PET. For CSF, Aβ-positivity (Aβ+) was defined as CSF Aβ42/Aβ40 < 0.071. This cutoff was previously established in the ALFA+ cohort using a Gaussian Mixture Model approach. The cutoff was defined as the mean minus 2 standard deviations (SD) of the non-pathologic Gaussian distribution of the CSF Aβ42/40 ratio [22]. Therefore, it corresponds to a degree of abnormality rather than a diagnostic cutoff. Aβ PET was assessed either by the CL scale or visual read. For the CL scale, we used the cutoff of 30 CL because it falls within the range of agreement with the visual read method in clinical populations (24–35 CL) and therefore marks established Aβ pathology [27, 33–36]. Alternatively, Aβ PET visual read was categorized as Aβ+ or Aβ- by a trained nuclear medicine physician and is the outcome used for clinical purposes and, typically, to recruit participants for anti-Aβ drug trials. Finally, we also used the range of 20–40 CL criteria because it is currently being used in preclinical AD clinical studies [18, 37] and can be informative for them.

We defined the group of participants with a low burden of Aβ pathology, referred to as the “low burden” group, using a combination of the aforementioned biomarkers cutoffs (Table 1):

- **Definition 1**: the low burden group was defined as those participants having a positive CSF Aβ42/Aβ40 ratio and CL values below 30 in Aβ PET. This group was compared with the Aβ- group (negative CSF Aβ42/Aβ40 ratio and CL < 30).
- **Definition 2:** the low burden group was defined as positive CSF Aβ42/Aβ40 ratio and negative Aβ PET visual read, and was compared with the Aβ- group (negative CSF Aβ42/Aβ40 ratio and negative Aβ PET visual read).
- **Definition 3:** the low burden group was defined as CL values ranging from 20 to 40 in Aβ PET, and was compared with the Aβ- group (CL < 20).

**Table 1:** Aβ groups definitions

	Aβ-	Low burden	Aβ+
Definition 1	CSF- and CL < 30	**CSF+ and CL < 30**	CSF+ and CL > 30
Definition 2	CSF- and VR-	**CSF+ and VR-**	CSF+ and VR+
Definition 3	CL < 20	**20–40 CL**	CL > 40

“Low burden” refers to “low burden of Aβ pathology”. Abbreviations: *Aβ* amyloid-β, *CL* Centiloid, *CSF* cerebrospinal fluid, *VR* visual read

### Statistical analysis

CSF biomarkers and neuroimaging variables extreme values, defined by 3 times the interquartile range below the first quartile or above the third quartile, were excluded. The following number of extreme values were identified and excluded: 4 for p-tau, 2 for t-tau, 3 for NfL, 1 for neurogranin, 1 for GFAP, 9 for IL6, 1 for S100B, and 12 for α-synuclein. In the main text, we show all the analyses excluding these extreme values. In the Additional file (Table S2), we show the same analyses including them.

The normality of each biomarker distribution was assessed using visual inspection of the histogram and the Kolmogorov-Smirnov test. None of the biomarkers, except Aβ42/40 and sTREM2, followed a normal distribution and they were thus log_10_-transformed.

We performed a one-way analysis of variance (ANOVA) to assess differences in age, years of education, and MMSE performance between the Aβ-, low burden and Aβ+ groups, and a Pearson’s chi-squared test to test differences in the distribution of sex and *APOE*-ε4 genotypes. *APOE* genotype was binarized as *APOE*-ε4 carriers or non-carriers.

CSF biomarker levels and neuroimaging variables were compared between groups using a one-way analysis of covariance (ANCOVA), adjusting for the effect of age and sex. Hippocampal volume analyses were also adjusted by the estimated total intracranial volume (eTIV). All significant comparisons were followed by Dunnett-corrected post hoc pairwise comparisons, with the Aβ- group as the reference group.

All tests were 2-tailed, with a significance level of α = 0.05. We applied a false discovery rate (FDR) multiple comparison correction following the Benjamini-Hochberg procedure [38].

Statistical analyses were performed in SPSS IBM, version 20.0, statistical software and the open-source statistical software R. Figures were built using R.

### Standard protocol approvals, registrations, and patient consents

The ALFA+ study (ALFA-FPM-0311) was approved by the Independent Ethics Committee “Parc de Salut Mar”, Barcelona, and registered at ClinicalTrials.gov (Identifier: NCT02485730). All participating subjects signed the study’s informed consent form that had also been approved by the Independent Ethics Committee “Parc de Salut Mar”, Barcelona.

### Data availability statement

Due to participant’s privacy, individual-level data cannot be made publicly available. Researchers who wish to use data from the ALFA study must obtain approval from the ALFA study Management Team.

## Results

### Participants’ characteristics and low burden groups

We included 318 cognitively unimpaired, middle-aged (50–74 years old) participants from the ALFA+ cohort. We first compared the prevalence of the low burden group between the three definitions used, and it differed considerably: 88 (27.7%), 72 (23.0%), and 18 (5.66%) participants fulfilled low burden criteria for definitions 1, 2, and 3, respectively (Table 2 and Table S1 in Additional file).

**Table 2:** Participants’ characteristics

	Definition 1 (n = 318)				Definition 2 (n = 313*)				Definition 3 (n = 318)			
	Aβ-	Low burden	Aβ+		Aβ-	Low burden	Aβ+		Aβ-	Low burden	Aβ+	
	CSF- CL < 30 (n = 205, 64.5%)	CSF+ CL < 30 (n = 88, 27.7%)	CSF+ CL > 30 (n = 25, 7.86%)	***P*** value	CSF- VR- (n = 202, 64.5%)	CSF+ VR- (n = 72, 23.0%)	CSF+ VR+ (n = 39, 12.5%)	***P*** value	CL < 20 (n = 281, 88.4%)	20 - 40 CL (n = 18, 5.66%)	CL > 40 (n = 19, 6.0%)	***P*** value
Age, years	60.5 (4.32)	61.2 (5.14)	65.7 (2.64)^1,2^	**<0.0001**	60.5 (4.34)	60.8 (5.23)	64.9 (3.41)^1,2^	**<0.0001**	60.6 (4.61)	64.0 (3.53)^3^	65.5 (2.89)^1^	**<0.0001**
Female, n (%)	133 (64.9)	48 (54.5)	18 (72.0)	0.12	131 (64.9)	40 (55.6)	24 (61.5)	0.37	175 (62.3)	11 (61.1)	13 (68.4)	0.86
Education, years	13.4 (3.44)	13.7 (3.44)	12.2 (3.95)	0.19	13.6 (3.40)	13.5 (3.30)	12.5 (3.89)	0.20	13.5 (3.42)	13.3 (3.85)	12.5 (3.99)	0.53
MMSE	29.1 (0.98)	29.4 (0.91)	28.9 (1.09)	0.10	29.1 (0.95)	29.3 (0.90)	29.0 (1.09)	0.25	29.2 (0.95)	29.2 (1.04)	29.1 (1.03)	0.90
*APOE-*ε4 carriers, n (%)	85 (41.5)	70 (79.5)^1^	17 (68.0)	**<0.0001**	82 (40.6)	57 (79.2)^1^	28 (71.8)^3^	**<0.0001**	145 (51.6)	13 (72.2)	14 (73.7)	**0.049**
Centiloid (CL)	−4.58 (6.51)	7.06 (10.2)^1^	50.9 (12.6)^1,2^	**<0.0001**	−4.66 (6.51)	5.52 (9.40)^1^	36.3 (21.7)^1,2^	**<0.0001**	−2.17 (7.99)	28.2 (6.10)^1^	55.6 (10.6)^1,2^	**<0.0001**
CSF Aβ42/40	0.087 (0.009)	0.055 (0.010)^1^	0.040 (0.008)^1,2^	**<0.0001**	0.086 (0.009)	0.056 (0.011)^1^	0.045 (0.011)^1,2^	**<0.0001**	0.078 (0.016)	0.045 (0.010)^1^	0.041 (0.008)^1^	**<0.0001**

Data are expressed as mean (M) and standard deviation (SD) or number of participants (n) and percentage (%), as appropriate. One-way ANOVA followed by Tukey corrected post hoc pairwise comparisons was used to compared age, education, and MMSE performance and Pearson’s chi-squared test to compare sex and *APOE-*ε4 status between groups within each definition. CSF Aβ42/40 and CL were compared with an ANCOVA adjusted by age and sex followed by Tukey corrected post hoc pairwise comparison. The *P* values indicated in the last column refer to the group main effect. Significant *P* values are marked in bold. “Low burden” refers to “Low burden of Ab pathology”. Abbreviations: *Aβ* amyloid-β, *CL* Centiloid, *CSF* cerebrospinal fluid, *MMSE* mini-mental state examination, *VR* visual read

*In definition 2, we included 313 participants because 2 participants did not have Aβ PET visual read assessment available and 3 additional participants had a discrepant CSF and visual read assessment (negative CSF Aβ42/40 ratio but positive visual read). Participants with this biomarker profile do not fall in any of the definition 2 categories

^1^*P* < 0.0001 vs Aβ- group

^2^*P* < 0.0001 vs low burden group

^3^*P* < 0.01 vs Aβ- group

Participants with a low burden of Aβ pathology were significantly older than the Aβ- group participants only using definition 3, which uses Aβ PET but not CSF Aβ42/20 as a criterion (*P =* 0.007) (Table 2). In contrast, definitions 1 and 2, that include both Aβ PET and CSF Aβ42/20 as criteria, the low burden group mean age did not differ from that of the Aβ- group. For all definitions, participants in the Aβ+ groups were significantly older than those in the Aβ- group (*P* < 0.0001). In definitions 1 and 2, Aβ+ group participants were also older than those in the low burden group (*P* < 0.0001).

In definitions 1 and 2, the low burden group showed a higher prevalence of *APOE*-ε4 carriers than the Aβ- group (Table 2). In definition 3, there was a significant difference in the prevalence of *APOE*-ε4 among groups, but this did not survive the post hoc multiple comparisons (Table 2). Although definition 3 was only defined by Aβ PET CL, all the individuals in its low burden group (*n* = 18) had also abnormal CSF Aβ42/40 levels. No significant differences were found in the distribution of sex, education years, or cognitive performance among groups in any of the three definitions studied.

### CSF biomarker changes in individuals with a low burden of Aβ pathology

We next assessed whether participants with a low burden of Aβ pathology presented significant differences in AD CSF biomarkers’ levels, which would suggest that other AD-related pathophysiological processes different from Aβ pathology are already activated in this transitional stage.

**Fig. 1:**
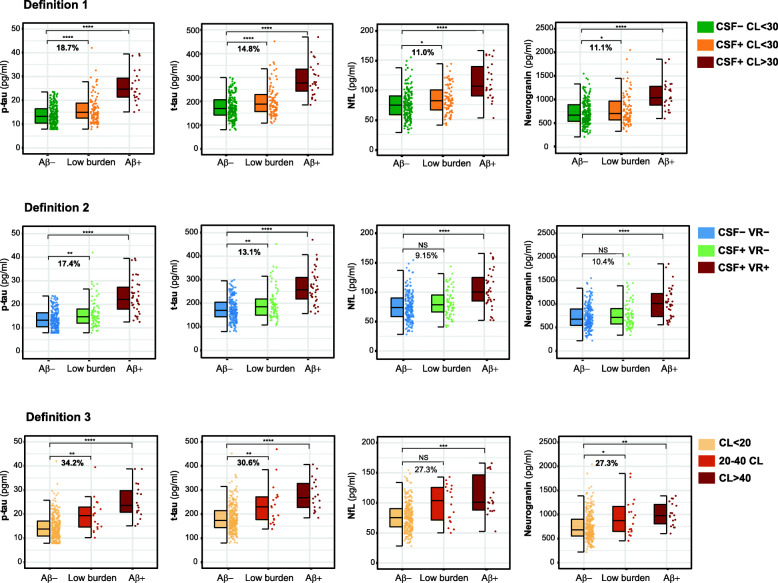
Comparison of CSF p-tau, t-tau, NfL, and neurogranin between Aβ groups. The boxplots depict the median (horizontal bar), interquartile range (IQR hinges), and 1.5 × IQR (whiskers). Group differences were assessed by a one-way analysis of covariance (ANCOVA) adjusted by age and sex, followed by Dunnett-corrected pairwise post hoc comparisons. We show the percentage of increase in the mean of each biomarker in the low burden group compared to the Aβ- group. The percentage is shown in bold if the difference is statistically significant. “Low burden” refers to “low burden of Aβ pathology”. *NS*, not significant. *P <0.05; **P <0.01; ***P <0.001; ****P <0.0001

We observed that CSF p-tau and t-tau were significantly increased in the low burden group compared to the Aβ- one in the three definitions (Table 3, Fig. 1). Moreover, CSF neurogranin was also significantly higher in the low burden group than in the Aβ- group in definitions 1 and 3 (Table 3, Fig. 1). Importantly, in definition 1, CSF NfL was also significantly increased in the low burden group, whilst a trend in the same direction is observed in definition 3 (*P =* 0.070). In the analyses including the extreme values (Table S2 in Additional file), the significant increase in CSF NfL in the low burden group in definition 1 was lost (*P* = 0.051).

**Table 3:** CSF biomarker levels by Aβ group

	Definition 1 (n = 318)				Definition 2 (n = 313*)				Definition 3 (n = 318)			
	Aβ-	Low burden	Aβ+		Aβ-	Low burden	Aβ+		Aβ-	Low burden	Aβ+	
	CSF- CL < 30 (n = 205, 64.5%)	CSF+ CL < 30 (n = 88, 27.7%)	CSF+ CL > 30 (n = 25, 7.86%)	***P*** value	CSF- VR- (n = 202, 64.5%)	CSF+ VR- (n = 72, 23.0%)	CSF+ VR+ (n = 39, 12.5%)	***P*** value	CL < 20 (n = 281, 88.4%)	20–40 CL (n = 18, 5.66%)	CL > 40 (n = 19, 6.0%)	***P*** value
p-tau (pg/ml)	13.9 (4.19)	16.5 (5.97)^1^	25.9 (6.94)^1^	**<0.0001**	13.8 (4.17)	16.2 (6.03)^2^	23.1 (7.40)^1^	**<0.0001**	14.6 (4.97)	19.6 (7.13)^2^	25.3 (7.08)^1^	**<0.0001**
t-tau (pg/ml)	176 (48.7)	202 (64.9)^3^	293 (71.6)^1^	**<0.0001**	176 (48.5)	199 (66.1)^2^	267 (75.1)^1^	**<0.0001**	183 (55.9)	239 (85.4)^2^	280 (65.8)^1^	**<0.0001**
NfL (pg/ml)	75.3 (23.6)	83.6 (23.4)^4^	114 (31.1)^1^	**<0.0001**	75.4 (23.7)	82.3 (22.9)	105 (31.4)^1^	**<0.0001**	77.4 (23.6)	98.5 (31.2)	112 (33.4)^3^	**0.0004**
Neurogranin (pg/ml)	722 (252)	802 (325)^4^	1103 (311)^1^	**<0.0001**	719 (251)	794 (330)	1019 (324)^1^	**<0.0001**	747 (282)	951 (402)^4^	1003 (239)^2^	**0.0009**
sTREM2 (ng/ml)	7.58 (1.93)	7.75 (2.15)	9.39 (2.83)^2^	**0.006**	7.57 (1.93)	7.74 (2.22)	8.83 (2.63)	0.053	7.63 (2.00)	8.84 (2.74)	8.79 (2.66)	0.065
YKL40 (ng/ml)	138 (44.8)	145 (49.8)	206 (58.5)^1^	**0.0002**	137 (44.6)	143 (51.4)	187 (58.5)^3^	**0.002**	140 (46.6)	170 (55.1)	200 (64.1)^2^	**0.004**
GFAP (ng/ml)	7.09 (2.13)	7.68 (2.32)	9.68 (2.58)^3^	**0.001**	7.08 (2.14)	7.67 (2.39)	9.00 (2.58)^2^	**0.011**	7.24 (2.22)	8.51 (1.91)	9.71 (2.74)^2^	**0.004**
IL6 (pg/ml)	3.86 (1.33)	3.81 (1.54)	3.97 (1.37)	0.33	3.87 (1.33)	3.90 (1.58)	3.72 (1.39)	0.89	3.86 (1.38)	3.66 (1.58)	3.94 (1.37)	0.56
S100B (ng/ml)	0.98 (0.20)	1.06 (0.26)	1.07 (0.25)	0.054	0.98 (0.20)	1.05 (0.25)	1.09 (0.27)	**0.044**	1.00 (0.21)	1.16 (0.33)	1.03 (0.18)	0.093
α-synuclein (pg/ml)	189 (80.9)	190 (62.5)	249 (87.3)^2^	**0.005**	189 (81.2)	184 (60.9)	239 (79.2)^3^	**0.001**	190 (76.6)	210 (82.3)	237 (85.1)	0.069

Data are expressed as mean (M) and standard deviation (SD). One-way ANCOVA adjusted by age and sex, followed by Dunnett-corrected post hoc comparisons, was used to compare CSF biomarker values between groups. The *P* values indicated in the last column refer to the group main effect. Significant *P* values are marked in bold. All *P* values remained significant after FDR multiple comparison correction, except S100B in definition 2 (*P =* 0.055). “Low burden” refers to “low burden of Aβ pathology”. Abbreviations: *Aβ* amyloid-β, *CL* Centiloid, *CSF* cerebrospinal fluid, *p-tau* phosphorylated tau, *t-tau* total tau, *NfL* neurofilament light, *S100B* S100 calcium-binding protein B, *sTREM2* soluble triggering receptor expressed on myeloid cells 2 (TREM2), *GFAP* glial fibrillary acidic protein, *IL6* interleukin 6, *VR* visual read, *YKL40* Chitinase-3-like protein 1

*In definition 2, we included 313 participants because 2 participants did not have Aβ PET visual read assessment available and 3 additional participants had a discrepant CSF and visual read assessment (negative CSF Aβ42/40 ratio but positive visual read). Participants with this biomarker profile do not fall in any of the definition 2 categories

^1^*P* < 0.0001 vs Aβ- group

^2^*P* < 0.01 vs Aβ- group

^3^*P* < 0.001 vs Aβ- group

^4^*P* < 0.05 vs Aβ- group

In definition 2, CSF neurogranin and NfL also showed a trend to increased values in the low burden group, but did not reach statistical significance (*P =* 0.081 and *P* = 0.080 for CSF neurogranin and NfL, respectively) (Table 3, Fig. 1).

Importantly, the magnitude of the changes in CSF biomarkers between the low burden and the Aβ- group differed considerably between definitions. In definition 3, the magnitude of the differences was always higher than those of definitions 1 and 2 (Fig. 1). In the case of CSF p-tau, the magnitude of change ranged from 17.4% (definition 2) to 34.2% (definition 3). For CSF t-tau, it ranged from 13.1% (definition 2) to 30.6% (definition 3). Group differences in CSF neurogranin ranged from 10.4% (definition 2) to 27.3% (definition 3), and those of CSF NfL from 9.15% (definition 2) to 27.3% (definition 3) (Fig. 1). This suggests that the low burden groups in definitions 1 and 2 capture a population with lower Aβ accumulation (as shown by their mean CL levels), which may suggest an earlier disease stage in the Alzheimer’s *continuum* than that in definition 3.

As expected, there was an increase in several CSF biomarkers in the Aβ+ group compared to the Aβ- one. CSF p-tau, t-tau, NfL, neurogranin, YKL40, and GFAP were increased in the Aβ+ group compared to the Aβ- group in the three definitions (Table 3; Fig. 1; Fig. S1 in Additional file).

### Structural brain changes associated with a low burden of Aβ pathology

We assessed whether a low burden of Aβ pathology was also associated with structural brain changes. Unlike the CSF biomarker results, there were no differences in the AD signature MRI Meta-ROI composite of the low burden and Aβ+ groups compared to the Aβ- groups in any of the three definitions. However, when we looked at the individual regions of interest that compose the AD signature, the low burden group in definition 2 showed a greater cortical thickness in the right fusiform gyrus compared to the Aβ- group (*P* = 0.049). In definition 2, there was also a trend to group differences in the middle temporal region, but it did not survive post hoc multiple comparisons (Table S3 in Additional file).

Among the other individual regions of interest analyzed, we found an increase in cortical thickness of the left bankssts in individuals with a low burden of Aβ pathology in definition 2 compared to Aβ- ones (*P* = 0.028). A trend to the same result was observed in definition 1 (group main effect *P* = 0.068). Noteworthy, these differences were not observed in the Aβ+ group (Table S3 in Additional file).

### Brain metabolism changes associated with a low burden of Aβ pathology

We next explored whether a low burden of Aβ pathology was associated with changes in brain metabolism as measured by FDG PET. Analysis of the AD signature FDG PET Meta-ROI composite showed no significant changes in the low burden group compared to the Aβ- in any of the three definitions. In contrast, Aβ+ individuals in definition 2 had a higher metabolism compared to Aβ- ones (*P* = 0.029) (Table S4 in Additional file).

We next analyzed the individual regions included in the Meta-ROI composite. In definition 1, there was a trend to a higher metabolism in the left angular gyrus in the low burden group compared to the Aβ- one (*P* = 0.066). In definition 2, this increase in metabolism in the angular gyrus was bilateral and significant in the Aβ+ group but not in the low burden group (Table S4 in Additional file). No significant group differences were found in any region studied in definition 3.

All significant results in the MRI and FDG PET analyses did not survive FDR multiple comparison correction (Table S3 and S4 in Additional file) and should therefore be interpreted as exploratory.

## Discussion

The main finding of our study is that individuals with a low burden of Aβ pathology, and before overt Aβ deposition is present, already show typical AD pathophysiological changes. Specifically, we found that the main AD CSF biomarkers, reflecting tau, synaptic, and neurodegenerative changes, are already altered at this stage, whilst structural and functional brain changes are still minimal or absent.

The long preclinical stage of the Alzheimer’s* continuum* includes a transition from the complete absence of pathology, followed by incipient subtle Aβ pathology and eventual overt Aβ and tau pathology [5]. In our study, we examined those individuals with a low burden of Aβ pathology, that is, individuals below the Aβ PET positivity typical thresholds but with some signs of subtle Aβ changes (shown by either CSF or PET Aβ biomarkers), probably reflecting a stage when Aβ pathology is emerging. The choice of the term “low burden of Aβ pathology” to define this stage was not arbitrary. This term objectively describes the biomarker findings, stresses the transitional nature of these initial Aβ-related changes, and it does not have temporal connotations (as the terms “emerging” or “incipient” pathology may have). Herein, we defined this low burden stage in three different ways. Two of them (definition 1 and definition 2) were based on the mismatch between CSF and PET Aβ-positivity, which reflects an early stage of Aβ dysregulation when CSF biomarkers have started to change but Aβ PET is still not positive [39, 40]. For Aβ PET classification, we used two different criteria: the CL scale, usually used for research purposes [26], and the visual read, usually used in the clinical setting. Definition 3 was merely based on a CL values window, which includes participants who are not above the threshold for Aβ positivity but show intermediate level of Aβ pathology in Aβ PET. By using different definitions of the low burden of Aβ pathology, we intended to confirm the consistency of our results and, at the same time, to assess definitions that may be or are already being used in studies at this early stage [18, 37]. Noticeably, the prevalence and the demographic characteristics of the low burden group are considerably different depending on the definition used, an issue that should be taken into consideration when designing studies at this stage.

Our most consistent finding was that some AD CSF biomarkers change as early in the *continuum* as there is evidence of a low Aβ pathological burden. Specifically, we observed an increase in CSF biomarkers reflecting tau pathology (p-tau), synaptic dysfunction (neurogranin), and neurodegeneration (t-tau and NfL). Nevertheless, there are some differences depending on the definition used. The low burden group as defined by definition 1 (i.e., CSF Aβ positive and CL < 30) shows significant changes in CSF p-tau, t-tau, neurogranin, and NfL. This is followed by definition 3 (i.e. CL 20–40), with significant increases in CSF p-tau, t-tau, neurogranin, and a tendency to increased CSF NfL. To note, the CSF biomarkers changes in definition 3 were those with the highest magnitude. Finally, the low Aβ pathology burden group in definition 2 (i.e., CSF Aβ positive and visual read negative) had increased CSF p-tau and t-tau but only a tendency to increased CSF neurogranin and NfL. In respect to this, the definition of visual read positivity in our sample is reached most probably at an equivalent CL value considerably lower than 30, within the range of 12–20 CL [41] [Lyduine Collij; *Alzheimer’s Association International Conference* 2020]. Therefore, definition 2 low Aβ pathology burden group would encompass the earliest of these three definitions of Aβ pathology, when only tau-related CSF biomarkers start to increase. Overall, these results are consistent with those showing that levels of Aβ pathology below the generally used Aβ PET thresholds can be biologically and clinically meaningful and predict subsequent tau pathology or cognitive decline [13, 14].

We also assessed possible structural and functional changes in individuals with a low burden of Aβ pathology. We investigated the structural MRI and FDG PET AD signatures as well as other areas of interest known to change early in AD [18, 31, 32]. The results were not as clear as those of the CSF biomarkers. We did not observe a significant decrease in cortical thickness or brain metabolism in any of the regions of interest in neither the low burden nor the Aβ+ groups. This probably reflects the early stage of our population in terms of the absence of Aβ-downstream structural and functional consequences (e.g., reduction of brain volume and decreased metabolism), which would be expected in later stages of the *continuum*. On the contrary, our results suggest a subtle trend to increased cortical thickness in the group of participants with a low burden of Aβ pathology (definition 2) but not in the Aβ+ group, especially in the bankssts and the right fusiform gyrus. This result is in line with previous findings that showed a nonlinear relationship between Aβ and cortical thickness, so that there is thickening in the bankssts region in cognitively healthy elderly individuals with intermediate CSF Aβ values [32]. Similarly, we observed a slight trend to an increased glucose metabolism, especially in the angular gyrus, associated to Aβ pathology.

The main reason underlying the differences in CSF and neuroimaging biomarker changes between the different low burden group definitions is that they probably capture different stages in the Alzheimer’s *continuum*. Definitions including CSF biomarkers (definitions 1 and 2) probably reflect earlier stages, while an approach solely defined by Aβ PET (definition 3) may reflect a later stage. This explains why definition 3 has the highest magnitude of changes in CSF biomarkers. Of note, all individuals of the definition 3 low burden group have also abnormal CSF Aβ42/40. In contrast, definition 2 low burden group may capture an earlier stage, as shown by its higher CSF Aβ42/40 ratio and lower CL values, and that could explain why there are mild increases in cortical thickness. This is an important consideration when designing an interventional study. If the study includes participants in the very early stage of the *continuum* (as apparently occurs in definition 2 of our study), non-monotonic changes in neuroimaging biomarkers should be considered. We should however acknowledge that there are other factors that may underlie the different results that we found between definitions as well as their different prevalence. CSF-based definitions may be also influenced by decreased CSF production or clearance with age or the fact that there are lower/higher Aβ producer individuals [42–44]. Still, the use of CSF Aβ42/40 ratio, instead of CSF Aβ42, accounts, at least partially, for these factors [45]. We also should keep in mind that this low burden group is a transitory category, and its prevalence may be also affected by age and prevalence of *APOE-ε*4 [46]. Remarkably, our cohort includes middle-aged healthy individuals, when Aβ pathology most likely starts and with a low prevalence of other co-morbidities (e.g., vascular risk factors, neuronal injury due to other causes) that may affect the biomarker results. Also, the prevalence of *APOE-ε*4 carriers was similar between the low burden groups of the three definitions.

### Strenghts and limitations

Our study is not free of limitations. It is a cross-sectional study and longitudinal studies assessing the evolution of Aβ pathology and Aβ-downstream pathological cascade are crucial. We presume that the low burden group represents an incipient stage of the disease and many of the individuals belonging to this group will most likely progress in the *continuum*. This is probably a heterogeneous group and it needs to be investigated what other factors may influence the eventual progression of these individuals to symptomatic stages. Moreover, we acknowledge that there are differences in the number of individuals in each low burden definition, which precludes statistical comparisons between them. Finally, none of the neuroimaging analyses survived FDR multiple comparisons correction and should therefore be interpreted cautiously. Nevertheless, our study has several strengths. First, the ALFA+ cohort encompasses middle age cognitively unimpaired individuals at higher risk of AD and therefore is an optimal population to study pathophysiological pathways emerging in the earliest stage of the Alzheimer’s *continuum.* Second, we have defined the low burden of Aβ pathology in three different ways, consistently observing similar results in the three of them. Finally, this is a multimodal study including a wide range of CSF biomarkers, as well as brain structure and metabolism markers.

## Conclusions

We show that there are biologically meaningful effects in individuals with a low burden of Aβ pathology, when structural and functional changes are yet either very subtle or absent. It still remains unanswered whether there is a therapeutic window in this very early stage (when Aβ probably starts to emerge and Aβ-related tau pathology and synaptic dysfunction progressively arise), and a later stage, still within preclinical Alzheimer, when neurodegeneration and downstream consequences in structural and functional neuroimaging measures occur. Yet, our findings suggest that there might be a window of opportunity for AD prevention that starts even before Aβ PET is positive using the typical thresholds. Following the idea that acting very early in AD is a priority, our results support the notion that individuals with even subtle Aβ changes should also be considered for inclusion in early prevention or intervention trials.

## Supplementary Information

**Additional file 1: Table S1.** Confusion table of group prevalence for each low burden definition. **Table S2.** CSF biomarker levels by Aβ group including CSF biomarkers extreme values. **Table S3.** Structural MRI measurements by Aβ group. **Table S4.** FDG PET measurements by Aβ group. **Fig. S1.** Comparison of all CSF biomarkers between Aβ groups.

## Data Availability

The data that support the findings of this study are available from the corresponding author, upon reasonable request.

## Abbreviations

Aβ: Amyloid-β
AD: Alzheimer’s disease
CSF: Cerebrospinal fluid
CL: Centiloid
PET: Positron emission tomography
p-tau: Phosphorylated tau
t-tau: Total tau
NfL: Neurofilament light
sTREM2: Soluble triggering receptor expressed on myeloid cells 2 (TREM2)
S100B: S100 calcium-binding protein B
GFAP: Glial fibrillary acidic protein
IL6: Interleukin 6
VR: Visual read
YKL40: Chitinase-3-like protein 1
