# Supplementary material for: Cognitively unimpaired individuals with a low burden of Aβ pathology have a distinct CSF biomarker profile
Source: Alzheimers Res Ther. 2021 Jul 27;13:134. doi: 10.1186/s13195-021-00863-y (PMC8314554; doi:10.1186/s13195-021-00863-y)
Supplement: Supplementary file 1 — Additional file 1: Table S1. Confusion table of group prevalence for each low burden definition. Table S2. CSF biomarker levels by Aβ group including CSF biomarkers extreme values. Table S3. Structural MRI measurements by Aβ group. Table S4. FDG PET measurements by Aβ group. Fig. S1. Comparison of all CSF biomarkers between Aβ groups. [file 13195_2021_863_MOESM1_ESM.docx]

**Additional file**

**Cognitively unimpaired participants with low burden of Aβ pathology have a distinct CSF biomarker profile**

**Authors:**

Marta Milà-Alomà, MSc, Mahnaz Shekari, MSc, Gemma Salvadó, MSc, Juan Domingo Gispert, PhD, Eider M. Arenaza-Urquijo, PhD, Grégory Operto, PhD, Carles Falcon, PhD, Natalia Vilor-Tejedor, PhD, Oriol Grau-Rivera, MD, PhD, Aleix Sala-Vila, PhD, Gonzalo Sánchez-Benavides, PhD, José Maria González-de-Echávarri, MD, Carolina Minguillon, PhD, Karine Fauria, PhD, Aida Niñerola-Baizán, PhD, Andrés Perissinotti, MD, PhD, Maryline Simon, PhD, Gwendlyn Kollmorgen, PhD, Henrik Zetterberg, MD, PhD, Kaj Blennow, MD, PhD, Marc Suárez-Calvet, MD, PhD^*^ José Luis Molinuevo, MD, PhD^*^ for the ALFA study

**Table of contents**

Table S1. Confusion table of group prevalence for each low burden definition 2

Table S2. CSF biomarker levels by Aβ group including CSF biomarkers extreme values 3

Table S3. Structural MRI measurements by Aβ group 5

Table S4. FDG PET measurements by Aβ group 7

Fig. S1. Comparison of all CSF biomarkers between Aβ groups 9

**Table S1. Confusion table of group prevalence for each low burden definition**

|  |  | **Definition 1** | | | **Definition 2** | | |
| --- | --- | --- | --- | --- | --- | --- | --- |
|  |  | CSF- CL<30 | **CSF+ CL<30** | CSF+ CL>30 | CSF- VR- | **CSF+ VR-** | CSF+ VR+ |
| **Definition 2** | CSF- VR- | 202 (64.5) | 0 | 0 |  |  |  |
|  | **CSF+ VR-** | 0 | 72  (23.0) | 0 |  |  |  |
|  | CSF+ VR+ | 0 | **15**  **(4.79)** | 24  (7.67) |  |  |  |
| **Definition 3** | CL<20 | 205 (64.5) | **76**  **(23.9)** | 0 | 202 (64.5) | **66**  **(21.1)** | **9**  **(2.88)** |
|  | **20-40 CL** | 0 | 12  (3.77) | 6  (1.89) | **0** | 6  (1.92) | **12**  **(3.83)** |
|  | CL>40 | 0 | 0 | 19  (6.0) | 0 | 0 | 18  (5.75) |

Confusion table showing the n (%) of participants in each category by each low burden definition. Bold numbers indicate discordance. “Low burden” refers to “Low burden of Aβ pathology”. Abbreviations: CL, Centiloid; CSF, cerebrospinal fluid; VR, visual read

**Table S2. CSF biomarker levels by Aβ group including CSF biomarkers extreme values**

|  | **Definition 1 (n = 318)** | | | | | **Definition 2 (n = 313*)** | | | | **Definition 3 (n = 318)** | | | |
| --- | --- | --- | --- | --- | --- | --- | --- | --- | --- | --- | --- | --- | --- |
|  | **Aβ-** | **Low burden** | **Aβ+** |  | **Aβ-** | | **Low burden** | **Aβ+** |  | **Aβ-** | **Low burden** | **Aβ+** |  |
|  | **CSF- CL<30** (n = 205, 64.5%) | **CSF+ CL<30** (n = 88, 27.7%) | **CSF+ CL>30** (n = 25, 7.86%) | ***P*-Value** | **CSF- VR-**(n = 202, 64.5%) | | **CSF+ VR-**(n = 72, 23.0%) | **CSF+ VR+** (n = 39, 12.5%) | ***P-*Value** | **CL<20** (n = 281, 88.4%) | **20-40 CL** (n = 18, 5.66%) | **CL>40** (n = 19, 6.0%) | ***P*-Value** |
| **p-tau (pg/ml)** | 13.9 (4.2) | 16.5 (6.0)^1^ | 28.9 (13.3)^2^ | **<0.0001** | 13.8 (4.2) | | 16.2 (6.0)^3^ | 25.1 (12.2)^2^ | **<0.0001** | 14.6 (5.0) | 21.0 (9.2)^1^ | 28.3 (14.5)^2^ | **<0.0001** |
| **t-tau (pg/ml)** | 176 (48.7) | 202 (64.9)^1^ | 308 (103)^2^ | **<0.0001** | 175 (48.5) | | 199 (66.1)^3^ | 277 (98.5)^2^ | **<0.0001** | 183 (55.9) | 239 (85.4)^3^ | 300 (110)^2^ | **<0.0001** |
| **NfL (pg/ml)** | 76.4 (28.4) | 85.4 (28.9) | 119 (37.5)^2^ | **<0.0001** | 766 (28.5) | | 84.5 (29.6) | 108 (36.4)^2^ | **0.0001** | 78.8 (28.8) | 98.5 (31.2) | 118 (41.4)^1^ | **0.0003** |
| **Neurogranin (pg/ml)** | 722 (252) | 802 (325)^4^ | 1154 (398)^2^ | **<0.0001** | 719 (251) | | 794 (330) | 1054 (387)^2^ | **<0.0001** | 747 (282) | 951 (402)^4^ | 1075 (392)^1^ | **0.0002** |
| **sTREM2 (ng/ml)** | 7.58 (1.93) | 7.75 (2.15) | 9.39 (2.83)^2^ | **0.006** | 7.57 (1.93) | | 7.74 (2.22) | 8.83 (2.63) | 0.053 | 7.63 (2.00) | 8.84 (2.74) | 8.79 (2.66) | 0.065 |
| **YKL-40 (ng/ml)** | 138 (44.8) | 145 (49.8) | 206 (58.5)^1^ | **0.0002** | 137 (44.6) | | 143 (51.4) | 187 (58.5)^3^ | **0.002** | 140 (46.6) | 170 (55.1) | 200 (64.1)^2^ | **0.004** |
| **GFAP (ng/ml)** | 7.2 (2.5) | 7.7 (2.3) | 9.7 (2.6)^3^ | **0.004** | 7.2 (2.6) | | 7.7 (2.4) | 9.0 (2.6)^4^ | **0.028** | 7.3 (2.5) | 8.5 (1.9) | 9.7 (2.7)^4^ | **0.010** |
| **IL6 (pg/ml)** | 4.1 (1.9) | 4.1 (2.2) | 4.5 (3.2) | 0.32 | 4.0 (1.8) | | 4.3 (2.3) | 4.1 (2.7) | 0.90 | 4.1 (2.0) | 4.0 (2.1) | 4.7 (3.6) | 0.39 |
| **S100 (ng/ml)** | 1.0 (0.2) | 1.1 (0.3)^4^ | 1.1 (0.3) | **0.018** | 1.0 (0.2) | | 1.1 (0.3) | 1.1 (0.3)^4^ | **0.019** | 1.0 (0.2) | 1.2 (0.3) | 1.1 (0.3) | 0.054 |
| **α-synuclein (pg/ml)** | 218 (186) | 226 (345) | 358 (350)^3^ | **0.002** | 210 (151) | | 228 (381) | 309 (288)^1^ | **0.002** | 222 (249) | 295 (371) | 302 (223) | 0.051 |

Data are expressed as mean (M) and standard deviation (SD). One-way ANCOVA adjusted by age and sex, followed by Dunnett-corrected *post hoc* comparisons was used to compare CSF biomarker values between groups. The *P*-values indicated in the last column refer to the group main effect. Significant *P*-values are marked in bold. All *P*-values remained significant after FDR multiple comparison correction. “Low burden” refers to “Low burden of Aβ pathology”. Abbreviations: Aβ42, amyloid-β 42; Aβ40, amyloid-β 40; CL, Centiloid; CSF, Cerebrospinal fluid; p-tau, phosphorylated tau; t-tau, total tau; NfL, neurofilament light; S100B, S100 calcium binding protein B; sTREM2, soluble triggering receptor expressed on myeloid cells 2 (TREM2); GFAP, glial fibrillary acidic protein; IL6, interleukin 6; VR, visual read; YKL40, Chitinase-3-like protein 1. *In definition 2 we included 313 participants because 2 participants did not have amyloid PET visual read assessment available and 3 additional participants had a discrepant CSF and visual read assessment (negative CSF Aβ42/40 ratio but positive visual read). Participants with this biomarker profile do not fall in any of definition 2 categories.

1 *P* < 0.001 *vs* Aβ- group

2 *P* < 0.0001 *vs* Aβ- group

3 *P* < 0.01 *vs* Aβ- group

4 *P* < 0.05 *vs* Aβ- group

**Table S3. Structural MRI measurements by Aβ group**

|  | **Definition 1**  **(n = 303)** | | | | **Definition 2**  **(n = 299*)** | | | | **Definition 3**  **(n = 303)** | | | |  |
| --- | --- | --- | --- | --- | --- | --- | --- | --- | --- | --- | --- | --- | --- |
|  | **CSF- CL<30**  (n = 192, 63.3%) | **CSF+ CL<30**  (n = 92, 30.4%) | **CSF+ CL>30**  (n = 19, 6.27%) | ***P-*Value** | **CSF- VR-**  (n = 190, 63.6%) | **CSF+ VR-**  (n = 70, 23.4%) | **CSF+ VR+**  (n = 39, 13.0%) | ***P-*Value** | **CL<20**  (n = 266, 87.8%) | **20-40 CL**  (n = 18, 5.94%) | **CL>40**  (n = 19, 6.27%) | ***P*-Value** |  |
| **AD signature Meta-ROI composite** | 2.41 (0.09) | 2.41 (0.09) | 2.40 (0.08) | 0.70 | 2.41 (0.09) | 2.42 (0.08) | 2.39 (0.08) | 0.21 | 2.41 (0.09) | 2.40 (0.06) | 2.40 (0.08) | 0.73 |  |
| Right entorhinal | 2.53 (0.31) | 2.54 (0.30) | 2.44 (0.25) | 0.29 | 2.53 (0.31) | 2.56 (0.31) | 2.46 (0.26) | 0.18 | 2.54 (0.31) | 2.45 (0.23) | 2.44 (0.24) | 0.16 |  |
| Left entorhinal | 2.59 (0.32) | 2.60 (0.26) | 2.61 (0.21) | 0.89 | 2.60 (0.32) | 2.63 (0.26) | 2.57 (0.25) | 0.36 | 2.60 (0.31) | 2.52 (0.23) | 2.61 (0.20) | 0.41 |  |
| **Average entorhinal** | 2.56 (0.25) | 2.57 (0.23) | 2.52 (0.21) | 0.47 | 2.56 (0.25) | 2.59 (0.23) | 2.51 (0.21) | 0.13 | 2.57 (0.25) | 2.48 (0.19) | 2.53 (0.20) | 0.17 |  |
| Right inferior-temporal | 2.30 (0.12) | 2.29 (0.12) | 2.29 (0.10) | 0.84 | 2.30 (0.12) | 2.29 (0.11) | 2.29 (0.12) | 0.89 | 2.30 (0.12) | 2.30 (0.09) | 2.30 (0.11) | 1 |  |
| Left inferior-temporal | 2.34 (0.11) | 2.34 (0.11) | 2.32 (0.11) | 0.79 | 2.34 (0.11) | 2.35 (0.11) | 2.33 (0.10) | 0.81 | 2.34 (0.11) | 2.31 (0.10) | 2.33 (0.12) | 0.52 |  |
| **Average inferior-temporal** | 2.32 (0.10) | 2.32 (0.09) | 2.31 (0.10) | 0.91 | 2.32 (0.10) | 2.32 (0.09) | 2.31 (0.10) | 0.95 | 2.32 (0.10) | 2.30 (0.07) | 2.31 (0.11) | 0.82 |  |
| Right middle-temporal | 2.53 (0.12) | 2.54 (0.13) | 2.51 (0.10) | 0.48 | 2.53 (0.12) | 2.55 (0.13) | 2.50 (0.11) | 0.13 | 2.53 (0.12) | 2.55 (0.11) | 2.50 (0.08) | 0.55 |  |
| Left middle-temporal | 2.50 (0.11) | 2.51 (0.11) | 2.48 (0.11) | 0.67 | 2.50 (0.11) | 2.52 (0.11) | 2.46 (0.11) | 0.089 | 2.51 (0.11) | 2.47 (0.10) | 2.48 (0.10) | 0.58 |  |
| **Average middle-temporal** | 2.52 (0.10) | 2.53 (0.11) | 2.49 (0.09) | 0.42 | 2.52 (0.10) | 2.54 (0.10) | 2.48 (0.10) | **0.038** | 2.52 (0.10) | 2.51 (0.09) | 2.49 (0.08) | 0.77 |  |
| Right fusiform | 2.35 (0.11) | 2.37 (0.10) | 2.33 (0.10) | 0.13 | 2.35 (0.11) | 2.38 (0.10)^1^ | 2.33 (0.10) | **0.044** | 2.35 (0.10) | 2.35 (0.09) | 2.34 (0.10) | 0.90 |  |
| Left fusiform | 2.40 (0.11) | 2.38 (0.10) | 2.39 (0.11) | 0.54 | 2.40 (0.11) | 2.39 (0.10) | 2.37 (0.11) | 0.27 | 2.39 (0.11) | 2.40 (0.08) | 2.38 (0.11) | 0.88 |  |
| **Average fusiform** | 2.37 (0.09) | 2.38 (0.08) | 2.36 (0.11) | 0.78 | 2.37 (0.09) | 2.38 (0.08) | 2.35 (0.09) | 0.16 | 2.37 (0.09) | 2.37 (0.07) | 2.36 (0.10) | 0.85 |  |
| **Other brain regions of interest** | | | | | | | | | | | | | |
| Right bankssts | 2.37 (0.13) | 2.38 (0.12) | 2.36 (0.11) | 0.68 | 2.37 (0.13) | 2.39 (0.13) | 2.35 (0.11) | 0.59 | 2.38 (0.13) | 2.37 (0.12) | 2.34 (0.11) | 0.86 |  |
| Left bankssts | 2.31 (0.11) | 2.34 (0.13) | 2.33 (0.13) | 0.068 | 2.31 (0.11) | 2.35 (0.13)^1^ | 2.30 (0.12) | **0.049** | 2.32 (0.12) | 2.30 (0.13) | 2.33 (0.13) | 0.59 |  |
| **Average bankssts** | 2.34 (0.10) | 2.36 (0.11) | 2.34 (0.08) | 0.17 | 2.34 (0.10) | 2.37 (0.11) | 2.33 (0.08) | 0.11 | 2.35 (0.10) | 2.33 (0.10) | 2.34 (0.07) | 0.96 |  |
| Right hippocampus | 3822 (373) | 3820 (360) | 3571 (358) | 0.13 | 3818 (373) | 3799 (354) | 3690 (393) | 0.14 | 3821 (374) | 3744 (281) | 3576 (384) | 0.40 |  |
| Left hippocampus | 3728 (347) | 3774 (360) | 3557 (410) | 0.72 | 3726 (348) | 3746 (354) | 3670 (410) | 0.69 | 3740 (355) | 3714 (362) | 3548 (383) | 0.71 |  |
| **Average hippocampus** | 3775 (344) | 3797 (344) | 3564 (375) | 0.31 | 3772 (345) | 3772 (338) | 3680 (390) | 0.30 | 3780 (348) | 3730 (303) | 3562 (379) | 0.50 |  |
| Right precuneus | 2.24 (0.09) | 2.24 (0.08) | 2.23 (0.07) | 0.68 | 2.24 (0.09) | 2.24 (0.08) | 2.22 (0.07) | 0.73 | 2.24 (0.08) | 2.24 (0.09) | 2.23 (0.08) | 0.79 |  |
| Left precuneus | 2.24 (0.08) | 2.23 (0.08) | 2.21 (0.07) | 0.75 | 2.24 (0.08) | 2.24 (0.08) | 2.21 (0.07) | 0.36 | 2.24 (0.08) | 2.24 (0.08) | 2.20 (0.07) | 0.41 |  |
| **Average precuneus** | 2.24 (0.08) | 2.23 (0.07) | 2.22 (0.07) | 0.94 | 2.24 (0.08) | 2.24 (0.07) | 2.21 (0.06) | 0.54 | 2.24 (0.08) | 2.24 (0.08) | 2.21 (0.07) | 0.72 |  |

Data are expressed as mean (M) and standard deviation (SD). The AD Signature Meta-ROI composite regions, the Banksst and the precuneus measurements are expressed as cortical thickness, whilst hippocampal measurements correspond to grey matter volume. Right (R) and left (L) areas have been analysed separately and averaged. One-way ANCOVA adjusted by age and sex, followed by Dunnett-corrected *post hoc* comparisons was used to compare neuroimaging measures between groups. The *P*-values indicated in the last column refer to the group main effect. Significant *P*-values are marked in bold. None of the significant associations survived FDR multiple comparison correction. Abbreviations: CL, Centiloid; CSF, Cerebrospinal fluid; Bankssts, banks of the superior temporal sulcus; VR, visual read. *303 participants had structural MRI available for definitions 1 and 3. In definition 2 we included 299 participants because 1 participant did not have amyloid PET visual read assessment available and 3 additional participants had a discrepant CSF and visual read assessment (negative CSF Aβ42/40 ratio but positive visual read). Participants with this biomarker profile do not fall in any of definition 2 categories.

1 *P* < 0.05 *vs* Aβ- group

**Table S4. FDG PET measurements by Aβ group**

|  | **Definition 1**  **(n = 318)** | | | | **Definition 2**  **(n = 313*)** | | | | **Definition 3**  **(n = 318)** | | | |
| --- | --- | --- | --- | --- | --- | --- | --- | --- | --- | --- | --- | --- |
|  | **CSF- CL<30**  (n = 205, 64.5%) | **CSF+ CL<30**  (n = 88, 27.7%) | **CSF+ CL>30**  (n = 25, 7.86%) | ***P-*Value** | **CSF- VR-**(n = 202, 64.5%) | **CSF+ VR-**(n = 72, 23.0%) | **CSF+ VR+** (n = 39, 12.5%) | ***P*-Value** | **CL<20** (n = 281, 88.4%) | **20-40 CL** (n = 18, 5.66%) | **CL>40** (n = 19, 6.0%) | ***P*-Value** |
| **AD signature Meta-ROI composite** | 1.76 (0.17) | 1.79 (0.16) | 1.80 (0.15) | 0.062 | 1.76 (0.17) | 1.78 (0.17) | 1.80 (0.13)^1^ | **0.037** | 1.77 (0.17) | 1.78 (0.13) | 1.80 (0.15) | 0.27 |
| Right angular | 1.62 (0.20) | 1.66 (0.18) | 1.66 (0.14) | 0.058 | 1.62 (0.20) | 1.64 (0.19) | 1.67 (0.14)^1^ | **0.019** | 1.63 (0.20) | 1.64 (0.15) | 1.66 (0.14) | 0.34 |
| Left angular | 1.72 (0.20) | 1.77 (0.19) | 1.77 (0.20) | **0.026** | 1.72 (0.20) | 1.76 (0.20) | 1.77 (0.17)^1^ | **0.027** | 1.74 (0.20) | 1.75 (0.15) | 1.77 (0.20) | 0.31 |
| **Average angular** | 1.67 (0.19) | 1.71 (0.17) | 1.71 (0.16) | **0.045** | 1.67 (0.19) | 1.70 (0.19) | 1.72 (0.15)^1^ | **0.025** | 1.68 (0.19) | 1.69 (0.15) | 1.71 (0.16) | 0.30 |
| Right middle inferior temporal gyrus | 1.39 (0.22) | 1.39 (0.24) | 1.38 (0.20) | 0.67 | 1.39 (0.22) | 1.38 (0.24) | 1.40 (0.24) | 0.51 | 1.39 (0.23) | 1.42 (0.22) | 1.38 (0.20) | 0.52 |
| Left middle inferior temporal gyrus | 1.81 (0.16) | 1.80 (0.15) | 1.80 (0.14) | 0.92 | 1.80 (0.16) | 1.79 (0.15) | 1.81 (0.14) | 0.48 | 1.80 (0.15) | 1.82 (0.16) | 1.80 (0.14) | 0.63 |
| **Average middle inferior temporal gyrus** | 1.60 (0.16) | 1.59 (0.16) | 1.59 (0.15) | 0.72 | 1.60 (0.16) | 1.58 (0.16) | 1.60 (0.16) | 0.39 | 1.60 (0.16) | 1.62 (0.15) | 1.59 (0.15) | 0.44 |
| **Bilateral posterior cingulate gyrus** | 2.10 (0.20) | 2.08 (0.18) | 2.13 (0.21) | 0.32 | 2.10 (0.20) | 2.08 (0.19) | 2.11 (0.17) | 0.57 | 2.09 (0.20) | 2.11 (0.13) | 2.13 (0.21) | 0.45 |

Data are expressed as mean (M) and standard deviation (SD) regional Standard Uptake value ratios (SVURr). One-way ANCOVA adjusted by age and sex, followed by Dunnett-corrected *post hoc* comparisons was used to compare neuroimaging measures between groups. Right (R) and left (L) areas have been analysed separately and averaged. The *P*-values indicated in the last column refer to the group main effect. Significant *P*-values are marked in bold. None of the significant associations survived FDR multiple comparison correction. Abbreviations: CL, Centiloid; CSF, Cerebrospinal fluid; VR, visual read. *In definition 2 we included 313 participants because 2 participants did not have amyloid PET visual read assessment available and 3 additional participants had a discrepant CSF and visual read assessment (negative CSF Aβ42/40 ratio but positive visual read).

1 *P* < 0.05 *vs* Aβ- group

**Figure S1**. Comparison of all CSF biomarkers between Aβ groups in low burden definition 1 (A), 2 (B) and 3 (C).


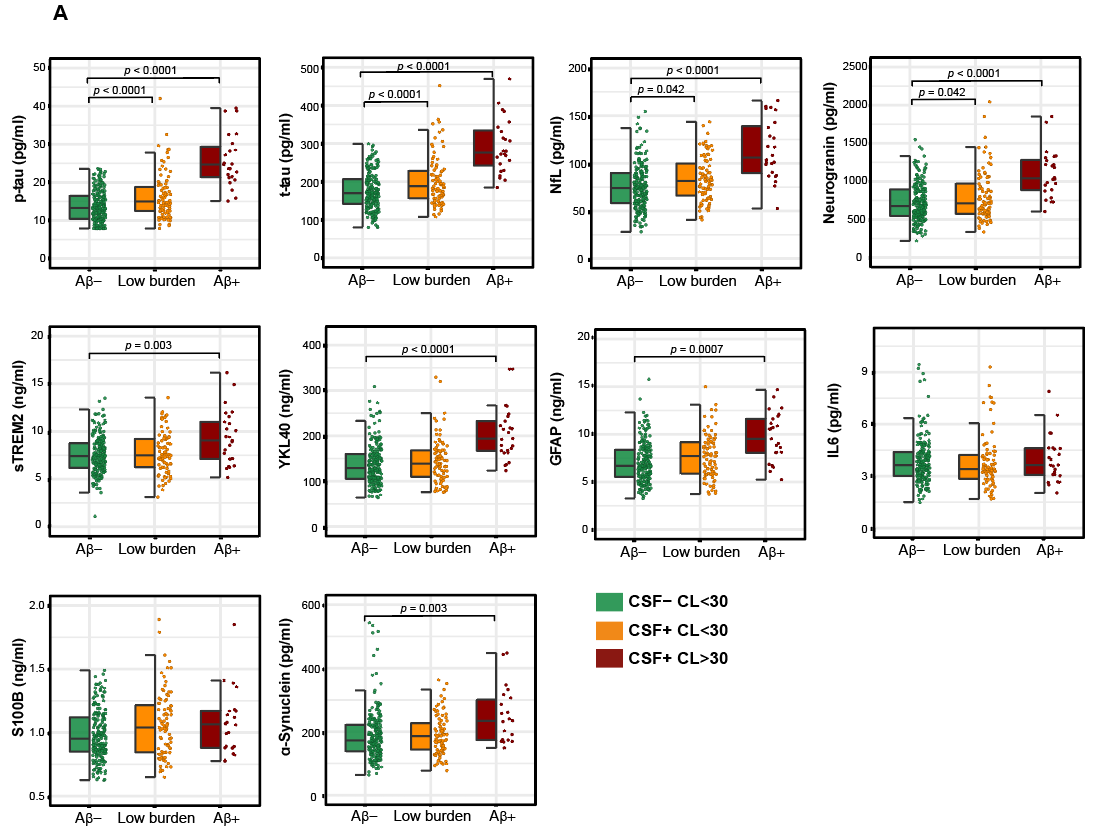


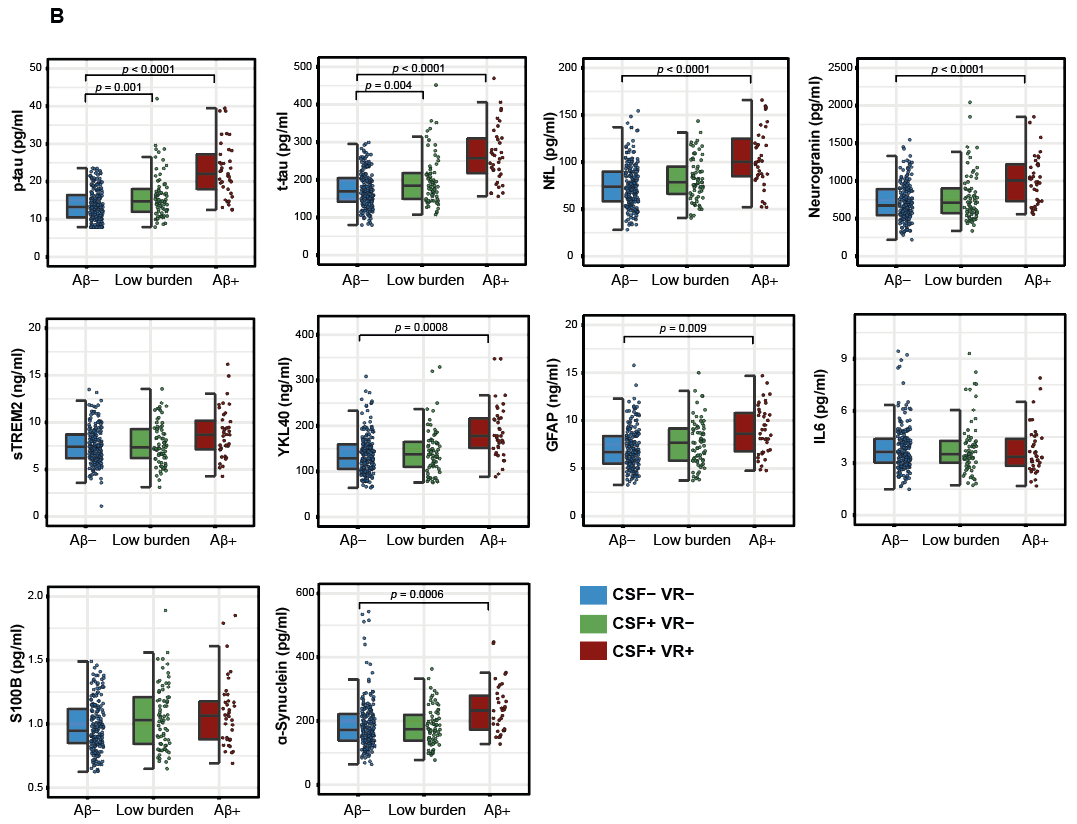


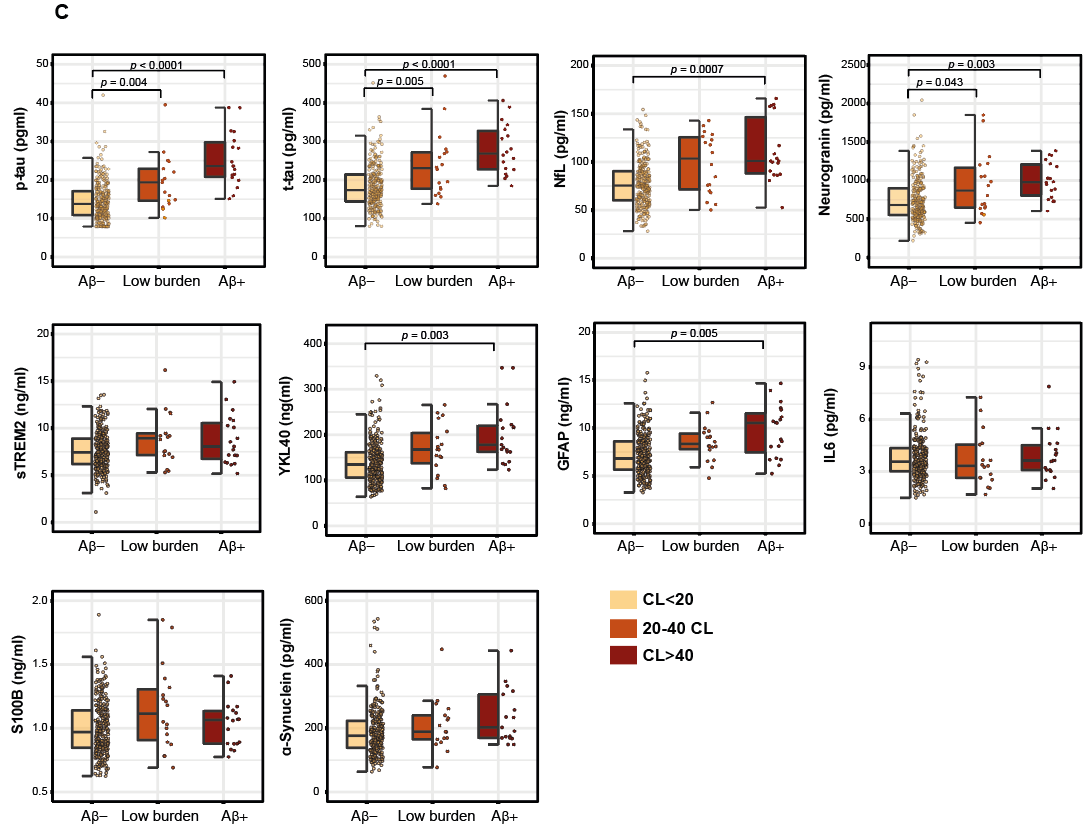


The boxplots depict the median (horizontal bar), interquartile range (IQR hinges), and 1.5 x IQR (whiskers). Group differences were assessed by a one-way analysis of covariance (ANCOVA) adjusted by age and sex, followed by Dunnet corrected pair-wise *post hoc* comparisons (Aβ- group as a reference group). “Low burden” refers to “Low burden of Aβ pathology”. Abbreviations: Aβ42, amyloid-β 42; Aβ40, amyloid-β 40; CL, Centiloid; CSF, cerebrospinal fluid; p-tau, phosphorylated tau; t-tau, total tau; NfL, neurofilament light; S100B, calcium binding protein B; sTREM2, soluble triggering receptor expressed on myeloid cells 2 (TREM2); GFAP, glial fibrillary acidic protein; IL6, interleukin 6; VR, visual read, YKL40, Chitinase-3-like protein 1
